# Parental Decision-Making on Childhood Vaccination

**DOI:** 10.3389/fpsyg.2018.00735

**Published:** 2018-06-13

**Authors:** Kaja Damnjanović, Johanna Graeber, Sandra Ilić, Wing Y. Lam, Žan Lep, Sara Morales, Tero Pulkkinen, Loes Vingerhoets

**Affiliations:** ^1^Laboratory for Experimental Psychology, Department of Psychology, Faculty of Philosophy, University of Belgrade, Belgrade, Serbia; ^2^Department of Psychology, Faculty of Philosophy, Christian-Albrechts-University Kiel, Kiel, Germany; ^3^Faculty of Social Sciences, School of Psychology, University of Kent, Canterbury, United Kingdom; ^4^Department of Psychology, Faculty of Arts, University of Ljubljana, Ljubljana, Slovenia; ^5^Faculty of Psychology, University of Basque Country, Bilbao, Spain; ^6^Department of Psychology and Logopedics, Faculty of Medicine, University of Helsinki, Helsinki, Finland; ^7^Department of Psychology, Faculty of Psychology and Neuroscience, University of Maastricht, Maastricht, Netherlands

**Keywords:** vaccine, involvement, vaccine hesitancy, immunization, health decisions, decision-making, parents, outcome bias

## Abstract

A growing number of parents delay vaccinations or are deciding not to vaccinate their children altogether. This increases the risk of contracting vaccine-preventable diseases and disrupting herd immunity, and also impairs the trust in the capacities of health care systems to protect people. Vaccine hesitancy is related to a range of both psychological and demographic determinants, such as attitudes toward vaccinations, social norms, and trust in science. Our aim is to understand those determinants in parents, because they are a special group in this issue—they act as proxy decision makers for their children, who are unable to decide for themselves. The fact that deciding to vaccinate is a socially forced choice that concerns a child's health makes vaccine-related decisions highly important and involving for parents. This high involvement might lead to parents overemphasizing the potential side effects that they know to be vaccine-related, and by amplifying those, parents are more focused on the potential outcomes of vaccine-related decisions, which can yield specific pattern of the outcome bias. We propose two related studies to investigate factors which promote vaccine hesitancy, protective factors that determine parental vaccination decisions, and outcome bias in parental vaccination intentions. We will explore demographic and psychological factors, and test parental involvement related to vaccine hesitancy using an online battery in a correlation panel design study. The second study is an experimental study, in which we will investigate the moderating role of parents' high involvement in the specific domain of vaccination decision making. We expect that higher involvement among parents, compared to non-parents, will shape the pattern of the proneness to outcome bias. The studies will be conducted across eight countries in Europe and Asia (Finland, Germany, Hong Kong, the Netherlands, Serbia, Slovenia, Spain, and the United Kingdom), rendering findings that will aid with understanding the underlying mechanisms of vaccine hesitancy and paving the way for developing interventions custom-made for parents.

## Introduction

One of the greatest public health challenges today concerns suboptimal vaccine uptake rates. In 2017, measles affected 21,315 people and caused 35 deaths, according to WHO's press release from 19 February 2018. “The surge in measles cases in 2017 included large outbreaks in 15 of the 53 countries in the (European) region. The highest numbers of affected people were reported in Romania (5,562), Italy (5,006), and Ukraine (4,767)” (World Health Organization, 2018). Greece, Germany, Serbia, the United Kingdom, Spain, Bulgaria, and France also experienced large outbreaks (World Health Organization, 2018). This is a result of suboptimal vaccine rates (World Health Organization, 2017a): in many areas, the coverage rates of common vaccines have decreased below 95% that is postulated as the minimum to herd immunity, the effective halting of the spread of measles and other vaccine-preventable diseases.

While some children cannot be vaccinated for medical reasons and in some areas vaccines are not readily available, a growing number of children are not vaccinated or are vaccinated late largely due to their parents' conscious decision (Pearce et al., 2008). The resistance to be vaccinated or to delay vaccinations despite having available vaccinating services, has been dubbed vaccine hesitancy (Luthy et al., 2009; Gowda and Dempsey, 2013; World Health Organization, 2014). Numerous interventions have been introduced to combat vaccine hesitancy, but many are lacking in success (Sadaf et al., 2013; Dubé et al., 2014; Pluviano et al., 2017). To better combat vaccine hesitancy and optimize interventions, factors associated with parents' decisions on vaccination need to be identified and investigated.

As such, vaccine hesitancy is a multi-layered phenomenon, related, amongst others, to various factors of social and psychological kind. Several studies examining various populations and different vaccines have found that vaccine hesitancy is related, amongst other, to prior beliefs about vaccinations (Smailbegovic et al., 2003; Dubé et al., 2014), perceived benefits of vaccines (Myers and Goodwin, 2011), attitudes toward vaccines (Pareek and Pattison, 2000; Mohd Azizi et al., 2017), whether the child has been previously vaccinated (Pareek and Pattison, 2000), previous experiences with vaccinations (Boes et al., 2017), socioeconomic status (Smith et al., 2004), number of children (Gust et al., 2005), and marital status (Smith et al., 2004). Despite the wide range of findings, the results of several systematic reviews suggest that there are still factors to be identified and further explored (Mills et al., 2005; Larson et al., 2014; Cobos et al., 2015).

The studies about vaccine hesitancy have often focused on parents, who are the key propagators of vaccine hesitancy and consumers of anti-vaccine influences, while the children are the key victims. For parents, vaccinating their children could mean that parents have to witness their child's discomfort and have to face potential potential side effects. At the same time, not vaccinating may lead to contracting vaccine-preventable diseases, potential prosecution in certain countries, enrolment refusal in some schools, disrupting herd immunity, etc. Parents may also face social pressure, such as pressure from health-care professionals (Evans et al., 2001), or other kinds of social pressure, e.g., to be experts on vaccination mechanism and therefore reliable and informed decision makers. In the contemporary context, parents are prompted to take an active role in their children's healthcare (Pyke-Grimm et al., 1999), which places heavy burden on the parent (Wagenaar et al., 1988).

This is especially true in the realm of intensive parenting; one of the most dominant parenting styles today (Arendell, 2000; Smyth and Craig, 2017). The term was coined by Hays (1996) to describe parenting style closely linked to the pressure felt by parents, mostly women, because of their responsibility for all childcare related tasks, children's outcomes (intellectual, social, emotional, and health-related), and their need to protect the child from any harm or disease. These needs, although very common for majority of parents, in highly individualistic societies outturn as the less communal worldviews, and research shows that intensive (salutogenic) parenting was an important rationale for refusing vaccines, as salutogenic parents have higher sense of advocacy and feel more capable of taking care for the children without expert intervention or vaccines (Reich, 2014; Ward et al., 2017).

On discussions about vaccinating their children, parents emphasize the purpose and safety of vaccination rather than the procedure itself (Salmon et al., 2005; Miton and Mercier, 2015). This parental decision is often accompanied by limited knowledge (Downs et al., 2008; Zingg and Siegrist, 2012), threatening campaigns (Ruiter et al., 2014; Stronach, 2015), societal norms (de Visser et al., 2011; Oraby et al., 2014), and official consent (Leask et al., 2011). Vaccine-hesitant parents thus differ from non-parents in their perception regarding the dangers of vaccines, risk of side effects, and protective benefits. Similarly, the perceived danger of vaccines is associated with the reluctance to vaccinate (Wilson et al., 2008), and it has been suggested that this can play an important role in parents' actual decision on mandatory childhood vaccination (Sporton and Francis, 2001).

This high-stake parental position regarding vaccinations is further complicated by the characteristics of the decision itself. Decisions, among themselves, differ depending on whom we are deciding for: ourselves or someone else (Zikmund-Fisher et al., 2006). People also use different strategies when deciding about other people compared to deciding about inanimate objects (Goldstein and Weber, 1995). Additionally, the importance of a decision differs according to its domain (see Meta-Decision-Making Model; Payne et al., 1993). Especially in health-related decisions, the importance of the decision skews decision-making processes and related phenomena, such as proneness to risky decisions (Wang, 1996a; Fagley and Miller, 1997; Kühberger, 1998; Hanoch et al., 2006; Markiewicz and Weber, 2013; Gummerum et al., 2014; Zimerman et al., 2014; Damnjanović and Gvozdenović, 2016), susceptibility to cognitive biases (McNeil et al., 1982; Wang, 1996b; Tanner et al., 2008) and effort of strategies (Edwards et al., 2001; Almashat et al., 2008).

Health-related decisions also differ according to the extent of their importance (Thompson, 2007), which affects the level of involvement decision makers put into a decision (Solomon et al., 2006). Different decisions range on a continuum from fairly routine to those that require extensive thought and have a high level of involvement (Solomon et al., 2006). The level of involvement in the same decision can differ between people (Arora and McHorney, 2000). However, some decisions (e.g., health-related decisions) are generally assumed to be important for the great majority of people (Solomon et al., 2006). Parental decision about a child's health is a special and extreme case of health-related decision (Zikmund-Fisher et al., 2006). It is also highly involving in terms of affect and expectation (Wroe et al., 2004). There is evidence suggesting differences, not only between parents and non-parents (Donovan and Jalleh, 2000), but also parents with children of different ages (Henrikson et al., 2017) regarding intentions to vaccinate and seek information on vaccination. Decomposing involvement and its influence on decision-making processes can help with undermining vaccine hesitancy through minimizing cognitive obstacles to reasoning, which stem from high involvement.

Parental decision on child vaccination is a specific case of health-related decision (Zikmund-Fisher et al., 2006) that is highly involving in terms of affect and expectation (Wroe et al., 2004). When discussing vaccination and immunization, the emphasis is on its purposefulness, potential side effects, and efficacy of vaccination (Salmon et al., 2005; Miton and Mercier, 2015). When normative, but also descriptive theory of decision-making is applied, decisions on vaccination can be analyzed using the decision matrix in which states of nature and alternative decisions are crossed to make cells with different outcomes (see Table 1). It can be stipulated that, when deciding on vaccination, people tend to place major weights on the outcomes, that is on the subjective perception of the outcomes. In other words, as well as some other health-related decisions, decisions on vaccination have an inherent feature of a stronger focus toward the outcome (Gellin et al., 2000; Freed et al., 2004, 2010; Gust et al., 2004; Miton and Mercier, 2015). To parents, the important issue while deciding is the outcome of this decision (e.g., well-being and health of their child) (Goldenberg, 2016). Evaluating prior decisions based on their outcomes is a tendency labeled as the outcome bias (Baron and Hershey, 1988). For instance, parents might overemphasize the immediate vaccine side effects, such as rashes or swelling, and use these side effects as justification to avoid vaccinating their child (Callender, 2016). In line with this, parents might judge the quality of the potential decision to vaccinate their child based on the consequences of this decision met previously by them or by the sources they are in contact with. Therefore, this decision is specific due to its explicit orientation toward the outcome (Gellin et al., 2000; Freed et al., 2004, 2010; Gust et al., 2004; Salmon et al., 2005).

**Table 1:** Decision matrix: vaccination case.

		**STATES OF NATURE**		
		**Vaccines are efficient and risk-free**	**Vaccines are efficient and low-risk**	**Vaccines are extremely dangerous**
Decisions	To vaccinate	Status quo	Status quo	High risk
	Not to vaccinate	High risk	High risk	Status quo

Understanding how both psychological and social factors relate to vaccine hesitancy is important for developing effective interventions. Therefore, the aim of the proposed research is to detect factors associated with and affecting the decisions of parents' regarding vaccination. To do that, we will conduct two separate but related studies. In study 1, socio-demographic and psychological variables will be tested for their connection with differences among parents when it comes to making vaccine-related decisions for their children. In study 2, the role of involvement in decisions regarding childhood vaccinations will be explored in more details. Specifically, we will study whether involvement will moderate the susceptibility to outcome bias with an experimental design.

## Study 1—correlates of intention to vaccinate

### Introduction

In study 1, we aim to explore the demographic and psychological factors that influence parents' vaccine hesitancy. As previously stated, vaccine hesitancy is related to a large range of attitudes, most notably to lower rates of compliance, which lead to drops in vaccination rates (Bloom et al., 2014). Our choice of correlates is in line with the framework of vaccine decision factors proposed by Gowda and Dempsey (2013), and it is important to acknowledge their interrelatedness. Since vaccination intention and hesitancy are multi-layered phenomena, chosen measures are narrowed to broadly cover the three following aspects: parent-specific factors (demographics, knowledge etc.), vaccine-specific factors (perceived vaccine safety and efficacy etc.), and external factors (values, norms, policies, requirements etc.).

#### Trust toward authorities

In the abundance of both affirmative and diminishing information on vaccination, the full picture is seldom easily available and individuals have a hard time forming their own opinions on the topic. Thus, argumentation must rely on evidence, which is accepted largely based on trust (Miton and Mercier, 2015). Trust in relevant actors (such as health professionals, pharmaceutical companies, law makers etc.) in the debate as well as general trust in science play an important role in vaccination decisions (Bedford, 2014; Jolley and Douglas, 2014; Camargo and Grant, 2015). However, this can be challenging as research repeatedly shows that some actors and science as a whole receive a low level of public trust (Lewandowsky and Oberauer, 2016). This can be caused due to high informational pluralism, rendering their argumentation on the topic irrelevant to the public.

Feelings of mistrust could also be part of a general feeling of unease about the complexity of modern society that forces us to rely on others to manage some parts of our lives (Hobson-West, 2007). According to Collins (2009), a general mistrust in science and scientists has enabled a paralyzing form of skepticism and scientific populism that denies the role of science and prompts anti-vaccination decisions.

Parents who have a positive view of the government are more likely to support vaccine policies, and perceive them as beneficial rather than restrictive of their personal freedom (Miton and Mercier, 2015; Highland, 2016). We expect parents who find official pro-vaccination authorities trustworthy to be less vaccine hesitant, show a higher intention to vaccinate, and a higher experience of freedom in the decision.

Previous studies have found that people with higher level of distrust toward authorities are more reluctant to rely on official sources of information (Freed et al., 2011). For that reason, we expect the relationship between level of trust and intention to vaccinate to be moderated by the type of sources consulted to make the decision. We expect level of trust toward authorities to predict the proportion of official sources used for the decision.

Finally, some studies have found that belief in conspiracy theories can predict distrust toward authorities (Darwin et al., 2011; Swami et al., 2011). We expect to replicate this result in the case of vaccine related conspiracy theories.

#### Perceived consensus and norms

Many vaccination decisions are influenced by parents' perception of others when making vaccination decisions (Gust et al., 2004; Leask and MacArtney, 2008; Gowda et al., 2012; Gowda and Dempsey, 2013). People in general tend to rely on consensus cues, because consensus, especially combined judgment of multiple experts, typically implies correctness (Van der Linden and Lewandowsky, 2015; Tom, 2017). However, there is a gap between the low level of scientific consensus perceived by the lay-public, and the actual level of consensus regarding the immunization and vaccination. The Gateway Belief Model proposed by Van der Linden et al. (2015) suggests that reducing the difference between people's subjective perception and the actual level of normative agreement among influential referents can lead to small yet important changes in key personal beliefs. Moreover, perceived scientific consensus has been identified as a key determinant in the public's opinion on, in some aspects equivalent, disputable topics (van der Linden et al., 2017). Due to high involvement aspects of vaccine-related decisions, we assume perceived scientific consensus plays a specific role, as it was the strongest predictor of the acceptance of the scientific arguments in other similar social issues (Lewandowsky et al., 2012). We expect parents who perceive stronger scientific consensus on the topic of vaccination to show less vaccine hesitancy and be more likely to vaccinate their children. We will thus test the correlation of perceived consensus, and the perception of risk with the decision on mandatory childhood vaccination (Wilson et al., 2008; Rolfe-Redding et al., 2012). We expect perceived consensus will correlate more strongly with the intention to vaccinate than perceived vaccination risks. Confidence in vaccines and vaccine-related decisions are also influenced by the individual's perception of societal norms and collective values, as well as their metacognitive perceptions about other groups' (e.g., health professionals) beliefs (Kennedy et al., 2011; Gowda and Dempsey, 2013; van der Linden et al., 2017). Numerous findings suggest normative information is rated as more trustworthy, less resilient to dismissal, and more influential than anecdotal cases (Carrico et al., 2011; Kahan et al., 2011; Lewandowsky et al., 2012; Rolfe-Redding et al., 2012), hence making it more likely to influence decision-making processes.

In situations where social norms are ambiguous, appealing to consensus and unity of norms tends to be more effective in persuading parents to vaccinate (Lewandowsky et al., 2012). Based on findings by Kahan et al. (2009) and Kahan (2010), we predict that the correlation between parents' intention to vaccinate (what we call adherence to the norm) and norms will be moderated by the perception of social consensus of given norms.

#### Freedom of choice, choice overload, and values

Choices are usually considered on a continuum from totally uninfluenced to the ones molded by formal and informal social norms. While norms play an important role in vaccination decisions (Gust et al., 2008; Brunson, 2013a,b), how we perceive those norms and how we perceive freedom when making the choice also influence the decision. This subjective perceived freedom is associated with choice overload in decision making processes (Lau et al., 2015). Decisions and decision-making processes can be exhausting and overwhelming, hence decision makers can find it difficult to retain all the necessary information needed to make an informed decision. We expect parents who experience lower levels of perceived freedom to have a higher tendency to adhere to perceived social norms. We also expect those individuals to be more likely to conform to authorities and to other stakeholders (health professionals, government etc.).

According to the cultural cognition of risk (Kahan and Braman, 2006; Kahan et al., 2010), the evaluation of riskiness is in line with values that we share as a culture. The operationalization of values regarding vaccination is a challenge, therefore we decided to use an indirect measure instead. We will measure participants' actively open-minded thinking style, a construct which was found to predict the tendency to acquire information in order to make competent decisions (Haran et al., 2013; Baron et al., 2015). We expect individuals who are more open-minded to be more likely to seek information from both sides of vaccine hesitancy spectrum (more diverse sources), be less affected by social norms, and show less vaccine hesitancy. The diversity in the sources of information will be rated by the proportion of official and informal sources.

#### Perception of danger

Threat perception has been widely used to encourage health-related actions such as vaccinations, but messages that increase risk perceptions are less effective than those increasing perceived effectiveness (Ruiter et al., 2001, 2014). However, parents are more likely to vaccinate or intend to vaccinate their children if they perceive the danger of not vaccinating (e.g., perceived vulnerability of their child contracting a certain disease) as high (Seeman et al., 2010; Healy and Pickering, 2011; Jolley and Douglas, 2014; Highland, 2016).

Health-related decisions often comprise of high levels of uncertainty and varying degrees of potential risk. Given that health professionals and other experts differ vastly in coping with uncertainty and risk taking (Grol et al., 1990), it is not surprising that parents are also under great strain when deciding. As stated before, parents often perceive vaccinating their children more risky than not vaccinating them, and as willingness to take risks is associated with making obligatory medical decisions (Grol et al., 1990), we expect to find the same connection in parents, with those less willing to take risks to be more vaccine hesitant.

#### Access to information

Perception of threat is mediated by access to information. Betsch et al. (2010) found that access to anti-vaccine sources of information increases perceived risks of vaccination. Furthermore, based on their risk perception, different parents trust different kinds of vaccine-related messages. Risk-oriented parents tend to favor statistical over anecdotal arguments, but those who are health-oriented tend to prefer the latter (Downs et al., 2008).

Moreover, knowledge has been identified as an important factor in shaping parents' decisions (Zingg and Siegrist, 2012). A higher number of sources of information has been related to a higher perceived level of knowledge in the frame of decision-making about vaccination (Downs et al., 2008; Rachiotis et al., 2010; Healy and Pickering, 2011; Brunson, 2013a), but the sources of their information can sometimes be problematic. Many parents reported seeking additional information, with most preferring to use the internet rather than consulting a doctor, and would use a general search engine instead of an official or medical website (Downs et al., 2008). Some parents were even found to reserve the decision to vaccinate until enough information was available to them (Highland, 2016). We expect participants who think they have enough access to information to be more likely to score on the extreme ends of the vaccine-hesitant spectrum (i.e., either very pro- or anti-vaccine), and those who are more exposed to anecdotal cases to be more vaccine hesitant. Additionally, in line with previous studies (Rachiotis et al., 2010; Jones et al., 2012; Brunson, 2013b) we expect the type of sources consulted (Official vs. Informal) to predict intention of vaccinate. Anecdotal cases (especially personal experiences) are one of the key forms of communication on the topic of vaccination, particularly among vaccine hesitant groups. This was supported by theoretical models such as the Cultural Attraction theory (Miton and Mercier, 2015), or the Fuzzy-Trace theory (Reyna and Brainerd, 1992; Reyna, 2008). Even vaccine concerns endorsed by a small but vocal group of individuals can heighten vaccine hesitancy in the community (Gowda and Dempsey, 2013). With vaccination being counter-intuitive in its nature (injecting antigens in an already healthy organism to remain healthy), anecdotal cases tap into our intuitive cognitive mechanisms, making individuals less likely to vaccinate. We expect parents that have been exposed to anecdotal cases of bad reactions to vaccines to show higher levels of vaccine hesitancy. However, as mentioned before, Risk vs. Health-orientation can moderate the effect of exposure to anecdotal cases (Downs et al., 2008). For that reason, we expect parents with a negative outcome focus to be more likely to be affetcted by the exposure to anecdotal cases.

### Methods

#### Design, sample, and procedure

Participants will be parents or primary caregivers of children that are of the recommended age to receive vaccinations from their corresponding countries across Europe and Asia (i.e., Finland, Germany, Hong Kong, the Netherlands, Serbia, Slovenia, and Spain). Participants must be (a) over 18 years old, and (b) a parent or care taker of at least one child under the age of 12 years (Salmon et al., 2005; Jones et al., 2012).

The sample size will be a minimum of 222 participants per country, based on a power analysis for an effect size of 0.15, and 20 predictors in a linear multiple regression model. The final sample size will not have a difference greater than 10% between each country. To make sure that there aren't any significant differences between the sample sizes of the different countries, if the sample of any country exceeds 10% of the mean, some participants will be randomly discarded until the criterion is met.

The questionnaire is programmed in JavaScript and will be administered online. To take part in the study, participants will need an electronic device with access to internet. The link with an unbiased invitation letter will be posted on different social media platforms, forums and websites, targeting a wide range of parents from the entire vaccine-hesitancy spectrum. Once the link is opened, participants will first be given a brief introduction of the study. Participants will then read and sign an informed consent, which states they are able to halt and withdraw from the study at any moment without providing a reason, and that they agree for their anonymized data to be analyzed for future publications. Participants will be asked to compose a unique identification code (consisting of their parents' initials, and month of birth), which will be used to identify their data if they decide to withdraw their data after completing the study. After that, participants will be asked to provide their demographic information and complete a battery of vaccination- and decision-making-related questions, with a total of 115 items (the complete battery is in Appendix 1). Participants will be able to leave items unanswered. The study will take ~25 min to complete, but will vary depending on participants' speed of responding. This procedure was ethically approved by the Institutional Review Board of the Faculty of Philosophy at the University of Belgrade.

#### Materials and measures

##### Choice of measures

Materials from online databases (PsycTESTS, DMIDI) were selected based on psychometric quality, fitness for parental context, translation feasibility, and the significance of usage in a variety of international institutions. Due to the complexity of our proposed model, and to avoid dropout due to the length of the final battery, we have adapted certain instruments and developed questions to assess specific constructs.

Additionally, data about each country will be gathered from different national, international and scientific entities, such as the percentage of vaccinated children in a country (World Health Organization, 2017b), number of physicians per 1000 inhabitants (World Health Organization, 2017a), health system quality (GBD 2015 Healthcare Access and Quality Collaborators, 2017) and about the vaccination programme in each of the countries (obtained from the corresponding governmental communications).

For the measurement of different constructs to be homogenous, all continuous variables will be measured with 7-point Likert-type scales. This is because most of the instruments used in the study originally included this type of scale, and the number of points in Likert-type scales do not influence its metric properties (Contractor and Fox, 2011).

##### Sociodemographic list

The sociodemographic data to be gathered includes participants' country of residence, age, gender, education (based on European Qualifications framework), marital status, number of children, age of children, the adherence to the official vaccination schedule of the child in the corresponding country, and if the participant themselves adhered to said vaccination schedule (Appendix 1). Subjective Socioeconomic Status (sSES) will rely on the level of education, and on participant's self-report regarding the “difficulty of the household to make ends meet” (Eurostat, 2017).

##### Intention to vaccinate

We will measure parents' willingness to vaccinate their children by their reported intention to vaccinate when presented with the option. To measure this, a single item will be used: “Would you at this time vaccinate your child according to the official vaccination schedule?” A version of this item, with an additional description: “Regardless if you are a parent or not,” was previously used by Stojkovic et al. (2017), who adapted it from the scales by Horne et al. (2015) and Opel et al. (2011). Parents will answer this item on a Likert-type scale ranging from “definitely yes” to “definitely not”. Participants' response to this question will be the dependent variable for this study.

##### Vaccination scales

To measure the perceived risk of (not) vaccinating may cause in children and society, we will use the vaccination scale developed by Horne et al. (2015). It is a 5-item scale that measures people's general attitude toward the vaccination (i.e., “Vaccinating healthy children helps to protect others by stopping the spread of diseases,” “I plan to vaccinate my children”). This test has proper psychometric features, including a high internal consistency (α = 0.84), and good predictive validity for past and future vaccination behavior, it has not been used in Europe so far.

Furthermore, the Vaccine Conspiracy Belief scale (Shapiro et al., 2016) will be added at this part. This scale contains 10 items, which examine the belief in the conspiracy theory that different entities try to hide the risk of vaccines. This shows the deception rather than the general attitude people have (Shapiro et al., 2016). An example of this item is: “The government is trying to cover up the link between vaccines and autism.” This scale has a very high level of internal consistency (α = 0.94) and is a good predictor of the willingness of parents to vaccinate their children.

##### Vaccine hesitancy

To identify vaccine hesitant parents, we will use Opel et al's. (2011) revised version of Parent Attitudes about Childhood Vaccines (PACV). The measure consists of 15 items, which are divided into three sub-domain scales: Safety and efficacy (α = 0.74), General attitudes (α = 0.84) and Behavior (α = 0.74). The measure has high internal and construct validity, and shows a statistically significant linear association between parents' total score on the 15-item PACV and their child's vaccination status (Opel et al., 2011). To increase the consistency of type of response along the scale and increase the sensitivity of the measure, we transformed some of the multiple-choice items to 7-point Likert type scales, in which each of the extremes represent the options that were included in the original scales. Finally, the item number 15 that referred to parents' trust toward their child's doctor, have been moved to the Trust Toward Authorities Scale (see Appendix 1 for detailed description of the battery).

##### Perceived freedom

The Experience of Freedom Measure (Lau et al., 2015) will be used to measure parents' perceived freedom when making vaccine-related decisions. Participants will rate 4-items (i.e., “I was able to choose what I wanted”) on a Likert-type scale. The measure has good internal consistency (α = 0.82).

##### Choice overload

Lau et al.'s (2015) Choice Overload scale will be used to measure the choice overload within the context of decision-making. Participants will be asked rate the extent to which they agree with 3 statements (i.e., “I felt overwhelmed by the decision”) on a Likert-type scale. This instrument shows good psychometric properties (α = 0.73) relative to the decision on whether to vaccinate their children.

##### Actively open-minded thinking

The Actively Open-Minded Thinking Beliefs (AOT) scale will be used to measure participants' beliefs on whether actively open-minded thinking is a desirable personal feature. The scale was originally developed by Stanovich and West (2007) and revised by Haran et al. (2013). We will use the revised version due to its shorter length (7 items) and adequacy for the general, adult population. Participants will rate how much they agree with given statements on a Likert type scale (i.e., “People should take into consideration evidence that goes against their beliefs”). The scale has been found to correlate with various measures of reflective thinking and good performance.

##### Trust toward authorities scale and sources of information

To measure the perceived credibility and the trust people have toward authorities, Jolley and Douglas' (2014) Trust toward Authorities scale will be used. The scale is built up out of items from previous scales. Participants will rate to what extent they trust corporations, national government, healthcare system, scientists, mainstream media, alternative media, social networks and their child's doctor on a Likert-type scale from strongly mistrust to strongly trust. They will also check all the sources they have used when making a decision about vaccination.

##### Availability of the relevant information

To assess whether parents believe they have enough information to make a solid decision regarding vaccinating their children, we will use one item from the General Health Styles survey by Gust et al. (2005): “I have access to all the information I need to make good decisions about immunization of my children.” Parents will answer on a Likert scale measuring the level of agreement.

##### Exposure to anecdotal cases

This variable will be measured through a single item adapted from the PACV scale by Opel et al. (2011) in a yes/no question format (i.e., “Have you ever heard of anyone who had a bad reaction to a shot?”).

##### Involvement in the vaccination decision

To measure personal involvement on the decision to vaccinate their children, we will include an item asking participants to rate their level of involvement. Additionally, we will ask if any other person is involved in the decision, and how many of them have been. For each of the additional people involved, participants will also be asked to indicate who that person is (the other parent of the child, another family member, a friend or any other person), indicate the gender, and rate the level of involvement of said person.

##### Perceived consensus, norms, and knowledge about vaccination

Additional Likert-type scale items will be used to assess participants' perceived scientific and social consensus about vaccination (i.e., “Is there a consensus among scientist about the safety of the vaccines?” “Is the vaccination an issue in your country?”), norms (i.e., “What do you think is the percentage of vaccinated children in your country?”), and knowledge (e.g., “Is vaccination mandatory in your country?”). The items are based on the items used by Van der Linden (2011) in research dealing with topics of perceived consensus and norms.

##### Passive risk-taking

We will measure participants' tendency of passive risk-taking using the Passive Risk-Taking Scale (Keinan and Bereby-Meyer, 2012). While risk mostly occurs during action, passive risk-taking can influence potential losses due to inaction. This test contains three subscales regarding risks that involve resources, medical issues and ethical issues. It has 25 items in total and uses a Likert-type rating scale in which participants will rate how likely they will act according to the statements (i.e., “Get vaccinated for the flu in the winter”). This scale has a high internal validity and reliability (α = 0.82).

##### Elaboration of potential outcomes

The Elaboration on Potential Outcomes (EPO, Nenkov et al., 2008) measure will be used to assess participants' tendencies to generate and evaluate possible positive and negative consequences of their behavior, and measure their attitudes toward risk-taking. The instrument consists of 13 items, which are divided into three subscales with high internal consistency: generation/evaluation (e.g., “I try to anticipate as many consequences of my actions as I can”; α = 0.88), positive outcome focus (e.g., “I keep a positive attitude that things always turn out all right.”; α = 0.87), and negative outcome focus (“I am often afraid that things might turn out badly”; α = 0.87). The measure was also found to have strong factor structure, high test-retest reliability and high predictive validity (construct of EPO is an important determinant of self-regulation).

#### Translation and the pilot studies

Every text in the study will be presented to participants in their own language: Traditional Chinese, Finnish, German, Spanish, Slovenian, Serbian, and Dutch. The questionnaires are constructed and written in English, then two separate translators for each of the seven languages translated (e.g., English to German) the battery. After that, back translations were compared with the original battery. The translators are native to the language they are translating to, and have at least a C1 level in the Common European Framework of Reference for Languages in said language. The process will be repeated until a third independent translator, native in English, considers that the original text and the back translation are equal in meaning.

The first pilot study will be conducted with the aim of testing the questionnaire framework and interaction with participant, and also to test psychometric characteristics of items and to eliminate those invalid. The battery is administered online in an English-speaking area, such as the UK, with a bigger sample that allows enough variability in the responses to the items to explore their functioning (i.e., 100 participants). We will assess item and subscale reliability and validity, and perform a factorial analysis.

Once the translation is prepared, we will conduct the second pilot study. The aim is to identify problems with items that may have appeared through the translation process. For example, items could be potentially ambiguous, unclear or misleading for participants (Ziegler et al., 2015). The battery will be administered to ~15 parents from each country and their responses will serve to adapt the problematic items and improve the battery.

### Proposed analysis

We will use statistical software (Matlab, R) for data analysis. We will analyse samples from each country separately because the differences in terms of language, legal framework in relation to vaccines, demographical characteristics and representativeness make the comparability between the different samples, and thus, their conjugate analysis, difficult (Ember et al., 1998).

**Figure 1:**
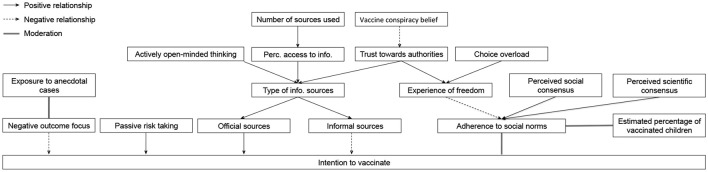
Proposed model of the factors of the intention to vaccinate.

For each sample, a factor analysis will be conducted to reduce the number of factors and control for interrelatedness of the variables. Two clusters of variables will be included: demographics (age, education level, number of children, mean age of children), and vaccine-related decision constructs (scores on scales on vaccination, vaccine hesitancy, perceived freedom of the decision, choice overload, actively open-minded thinking, trust toward authorities, availability of relevant information, perceived scientific consensus on vaccination, subjective estimation of percentage of children vaccinated on the country, perceived social consensus on vaccination, risk taking, and elaboration of potential outcomes). We expect the factor structure of each sample to be similar. With the reduced number of factors, a multivariate linear regression analysis will be conducted and the predictive power will be tested. The aforementioned factors will serve as predictors, and the parental intention to vaccinate (“I plan to vaccinate my child in accordance to the official vaccination schedule of my country”) will serve as criterion. All variables mentioned will also be included in a multivariate analysis of variance to see if there are differences between them depending on the level of adherence (total adherence, not completely total adherence, no adherence at all) to the official vaccination schedule of their children. We will test the proposed model of mediation (Figure 1) using regression analysis and compare it to a more complicated model with involvement as an additional factor.

For the open-ended question in which parents will detail their reasons for not absolutely adhering to the official vaccination schedule in their country, we will perform a qualitative analysis. After a pre-analysis, we will categorize answers based on the main reasons provided (e.g., medical reasons, considering vaccination risky etc.), and report the frequencies for each of them.

We will also check, using ANOVA, if there are differences in dependent variable (i.e., intention to vaccinate) according to participants' gender, marital status, while also considering whether vaccination is mandatory in their country of residence.

To check if the sources of information parents use to make vaccine-related decisions affect their intention to vaccinate, we will use ANOVA and *post-hoc* analysis.

### Anticipated results

The samples of this study will consist of data obtained in eight separate countries. This presents both challenges and possibilities. As some of the factors underlying vaccine hesitancy are context-specific and vary across time and place (Dubé et al., 2014), research in multiple countries is needed to understand vaccine hesitancy more fully on a local level. By analyzing our samples separately, we expect the study to contribute to knowledge on locally relevant factors related to vaccine hesitancy. However, the samples are unlikely to be representative in their respective countries, which limits the reliability of out conclusions on local, but not so much on the general level. Special care will also be taken to make sure that we sample people from all parts of the vaccine-hesitancy spectrum; the call for participants will be posted in different interest groups online along with general population call.

Given the sensitive nature of the topic, it is possible for the participants who participate in the study to have stronger convictions toward vaccines in one way or another, which might further decrease the representativeness of the vaccine hesitancy continuum—we want to include parents with various degrees of their hesitancy and strength of their convictions in order to avoid bipolarization. To decrease the effect of the topic on the motivation to participate, all the communication (e.g., invites, instructions) will be neutral in terms of referencing to potential harms and benefits of vaccination as well as any moral judgements of the decision.

Due to the wide range of phenomena of the present study, we hypothesize to find the following factors to reinforce the intent to vaccinate one's child: trust toward authorities, perceived social and scientific consensus, availability of relevant information, along with previously identified demographic characteristics and open-minded thinking. At the same time, we expect to confirm that vaccine hesitancy, perceived freedom, choice overload, the use of informal sources and susceptibility outcome bias serve as reinforces of delaying or omitting mandatory childhood vaccination. All our hypothesized connections can be seen in Figure 1.

## Study 2—outcome, not the decision maker, makes the choice

### Introduction

In order to further explore involvement as a factor, we will set up a second study. When people are making decisions about important issues (i.e., vaccine-related), the involvement in the decision and the aforementioned phenomenon of choice overload may be exacerbated. This is because ‘the costs associated with making the “wrong” choice, or even beliefs that “wrong” choices do indeed exist, are much more prominent, and substantial time and effort would be required for choosers to make truly informed comparisons among alternatives’ (Iyengar and Lepper, 2000). As the complexity of making choices rises, people tend to simplify their decision-making processes by relying on simple heuristics (Wright, 1975; Payne, 1982; Payne et al., 1988, 1993; Timmermans, 1993). To test whether involvement and choice overload moderate cognitive aspects of vaccination decision-making, we will use one of the empirically well-established cognitive biases as a litmus test, the outcome bias.

#### Outcome bias

People make and evaluate their own and other people's decisions every day, but different timings of these two cognitive processes lead to differences in available information (Baron and Hershey, 1988). All possible outcomes of a decision are mutually exclusive. In the moment of making a decision, the winning outcome and following consequences could not have been known to the decision maker, but these two factors are what the evaluator is familiar with. The outcome of a decision, however, should not be taken into account when evaluating the decision, because this information is irrelevant to the quality of the decision. The systematic tendency to evaluate the quality of a decision based on the outcome is called outcome bias (Baron and Hershey, 1988). People tend to use their knowledge about the outcome in an aforementioned, not logically justified manner (Allison et al., 1996; Gino et al., 2010), and judge the discernment and competence of decision makers based on it (Berg-Cross, 1975; Baron and Hershey, 1988; Lipshitz, 1989; Gino et al., 2010). However, there is no evidence on the relationship between the outcome bias and vaccine hesitancy, or outcome bias' connectedness to the level of parental involvement in the decision.

In the second study, involvement will be decomposed into three aspects: the situation decision makers are deciding about, decision makers' role, or who is a protagonist (parents vs. non-parents), and finally, low and high involvement health situations. Because the importance of the decision is related to susceptibility to cognitive biases, we expect that higher involvement among parents, compared to non-parents, will lead to greater proneness to making biased evaluations in health, but not in non-health related dilemmas (experiment 1). In a similar manner, parents will be more susceptible to outcome bias in both high and low involvement situations than non-parents (experiment 2). However, we expect that in dilemmas where the parents are the protagonists, the outcome bias will be stronger in non-parents (experiment 3).The results will help to understand the specific role of parents in vaccine decisions and will also contribute to the research about the relationship between vaccine hesitancy and cognitive biases, which at the current state is often hypothesized about, but lacking in papers.

### Methods

#### Sample

The second study will be conducted in Serbia. The sample will be comprised of parents and caregivers that participated in Study 1, and additionally an equal number of non-parents. Non-parents will be matched with parents who participated in Study 1 in terms of their age, gender and education. To estimate the sample size needed for the second study, special attention was paid to the minimization of Type II error. The analysis results of the test strength show that detecting a statistically significant outcome bias effect by a bivariate analysis of variance (at level *p* < 0.01) of the effect size of.7, as reported by Baron and Hershey (1988), for a sample of 20 subjects per experimental group amounts to 99.9%.

#### Design

This study will comprise of three experiments, by which we aim to decompose the parental involvement in a more detailed manner, to test if it moderates susceptibility to outcome bias in parents. Specifically, we will test if parents differ in biased reasoning from non-parents (experiment 1), do those two groups differ when judging about involving decisions (experiment 2), and finally do parents show higher understanding for other parents' decisions, in comparison with non-parents as judges (experiment 3). The design of all three experiments is mixed, 2 × 2 with two groups of participants, parents and non-parents.

Experiment 1 (Parenting and biased reasoning): 2 (levels of domain: health and non-health) × 2 (outcomes).

Experiment 2 (Parenting and involvement): 2 (levels of involvement) × 2 (outcomes)

Experiment 3 (Parenting and solidarity): 2 (levels of protagonists: parents and non-parents) × 2 (outcomes).

The independent variables, the domain, level of involvement in the decision, and protagonist will have two levels: health (e.g., Vitamin supplementation) and non-health domain (e.g., free time); low (e.g., should protagonist give a vitamin supplement to a child or not), and high involvement (e.g., should protagonist vaccinate a child or not); and who is making a decision (parent as protagonist or non-parent as protagonist). The outcome variable will have two levels: the positive and the negative outcome. By crossing two levels of each binary independent variable in all three experiments (domain, involvement, decision maker) with the parenthood of participants (parents and non-parents), four experimental situations will be formed: parents in, e.g., high involvement situation, parents in low involvement situation, non-parents in high involvement situation, non-parents in low involvement situation (see Table 2). Every situation will be formed with both outcomes (see Table 3). Equivalent design will be applied in all three experiments.

**Table 2:** Experimental situations in all three experiments.

	**Experiment 1 (domain)**		**Experiment 2 (involvement)**		**Experiment 3 (protagonist)**	
Factor levels	H	nH	hI	lI	P	nP
Parents	P × H	P × nH	P × hI	P × lI	P × x P	P × nP
Non-parents	nP × H	nP × nH	nP × hI	nP × lI	nP × P	nP × nP

*H, health-related; nH, non health-related; hI, high involvement; lI, low involvement; P, parent; nP, non parent*.

**Table 3:** Tasks for all three experiments.

	**Domain**		**Involvement**		**Protagonist**	
Factor levels	H	nH	hI	lI	P	nP
Positive outcome (+)	H+	nH+	hI+	lI+	P+	np+
Negative outcome (−)	H−	nH−	hI−	lI−	P−	nP−

*H, health-related; nH, non health-related; hI, high involvement; lI, low involvement; P, parent; nP, non parent*.

Each subject will participate in the procedure in two time slots, separated by 1 week (with opposite outcomes). For each experimental situation, a Latin square design will be used for randomizing the order of presentation of experimental tasks during two experimental sessions.

#### Stimuli and the third pilot study

The stimuli in this study will have the form of an evaluation task, principally used in judging and decision-making research paradigms. The text presented to the participant will consist of a prolog (a description of a situation that contains a dilemma), followed by explicitly stating which option the decision maker (DM; the protagonist of the presented situation) opted for, and the outcome of the decision. Outcomes of DM's decisions will be twofold: positive and negative. Participants' task will be to evaluate the presented decision by rating it on a scale from −3 to +3 [−3—the worst decision the DM could have made, +3—the best decision the protagonist (DM) could have made].

The third pilot study was conducted in Serbia, during December, with the aim to test items. The design was experimental, 2 (outcome) × 2 (involvement). By crossing factors of involvement and outcome, four categories of tasks were formed: high involvement with positive outcome, high involvement with negative outcome, low involvement with positive outcome, low involvement with negative outcome.

Participants were parents with pre-schoolers, younger than seven years old (*N* = 49, 73% female, mean age 34.88). Each participant was presented with 24 pairs of tasks consisting of a prolog, decision maker's decision, and the outcome of the decision in two time slots separated by two weeks. During the second session participants were presented with the same tasks but with outcomes opposite to those from the previous session. Participants' task was to evaluate the decision on a 10-point Likert scale (1 = the worst decision the DM could have made, 10 = the best decision the DM could have made).

Results showed that there was a statistically significant outcome bias detected on a sample as a whole [*F*_(1)_ = 283.239, *p* < 0.001]. Mean evaluation of stimuli with the positive outcome was 6.02 (*S* = 0.922), while the mean evaluation for stimuli with negative outcome was 3.56 (*S* = 0.834). The effect size coefficients were calculated for each pair of tasks. Cohen's D coefficients ranged from 0.223 to 1.604, with mean value of 0.957,

Based on these effect sizes, as well as on the analysis of participants' impressions about questions, a number of convenient stimuli will be selected for the Study 2 experiment.

#### Procedure

After successfully completing Study 1, the participants will be asked to take part in the experiment. The experiment will be conducted at the Faculty of Philosophy of University in Belgrade, in classrooms equipped with computers. The non-parent sample will be collected separately, from the available pool of participants from general population in database of partner institution. Participants will, again, have a self-generated personal code as their identification. Each subject will be provided with an introductory explanation for the following part of the study and given detailed instructions. Each participant will read a prolog (the situation), the decision the protagonist made and the outcome of the decision will be explicitly stated, and then participants will evaluate that decision. This process will be repeated twice. After finishing the second round, participants will be asked to give consent to store and use the data obtained in the current session, then they will be presented with a thank you note along with a reminder to visit the venue again in a week. They will also be asked to provide their e-mail address to enable us to send them a reminder, and, if they wish, a personal result, in comparison with both samples. After completing the task in the following week, participants will be asked to give consent again, presented with a thank you note, and the authors' email addresses if they have any inquiries regarding the experiment. Debriefing will be provided immediately, with the repeated emphasis that the situations described in the tasks are simulations and that they are not based on real data. The procedure was ethically approved by the Institutional Review Board of the Faculty of Philosophy at the University of Belgrade.

### Proposed analysis

The aim of this study is to investigate the moderating role of involvement in susceptibility to outcome bias.

Like in study 1, different samples will be treated separately. We will be using R and Matlab to conduct our analysis.

There is a possibility that participants might remember their answer from the first session and adapt their answer in the second session accordingly. To control this, we will use a two-way ANOVA. We will test whether the order of presentation of the stimuli, particularly whether the positive or negative outcome presented first, has an influence on the answers obtained during the second session.

To test our hypotheses, we will use a bivariate analysis of variance with repeated measures. As measures of the outcome bias, the difference between mean evaluations of decisions with positive and negative outcomes will be used to form 2 new variables for each of the 3 experiments. These new variables will then be used in the analysis. We will verify the assumption of standard distribution by conducting a Kolmogorov-Smirnov Test. If we cannot assume standard distribution, we will use the Wilcoxon signed rank test instead of an ANOVA. Effect sizes for each pair of tasks will be presented as Cohen's D.

## Joint discussion

With both studies we aim to investigate and come closer to understanding factors which influence vaccine hesitancy as a possible outcome of the vaccine-related decision-making processes, while focusing on testing the role of the involvement, as a potential underlying factor which skews this decision. Results of Study 1 will provide further insights into factors serving as reinforcements of the delay and omission of vaccination but also factors serving as reinforcements of the intention to vaccinate, as well as their interrelatedness. The results will provide insights into the construct of vaccine hesitancy that is currently lacking different stakeholders combating dropping immunization rates.

Moreover, if the Study 2 yields results consistent with our hypotheses, we will gain valuable proof that in terms of the extent and lengths of the decision-making processes regarding vaccination of their children, parents are indeed a special group, different from people without children, who do not have to face such dilemmas, and are therefore susceptible to different cognitive obstacles to reaching a decision to vaccinate.

With such knowledge it would be possible to draft interventions custom-made for parents aimed toward undermining their vaccine hesitancy by establishing better communication channels and better, more effective formation of relevant, informative and non-patronizing messages, addressing their personal dilemmas and fears with respect and understanding. Parents might not be afraid of vaccine-preventable diseases, but it seems they are afraid of vaccines – and the burden of the immunity of our herd lies on their shoulders.

## Ethics statement

This study was carried out in accordance with the recommendations of the Institutional Review Board of the Faculty of Philosophy of the University of Belgrade, who also ethically approved the proposed studies. All subjects gave written informed consent in accordance with the Declaration of Helsinki. The protocol was approved by the Institutional Review Board of the Faculty of Philosophy of the University of Belgrade.

## Author contributions

All authors listed have made a substantial, direct, and intellectual contribution to the work, and approved it for publication.

### Conflict of interest statement

The authors declare that the research was conducted in the absence of any commercial or financial relationships that could be construed as a potential conflict of interest.

## References

[B1] Allison S. T. Mackie D. M. Messick D. M. (1996). Outcome biases in social perception: implications for dispositional inference, attitude change, stereotyping, and social behavior. Adv. Exp. Soc. Psychol. 28, 53–93. 10.1016/S0065-2601(08)60236-1

[B2] Almashat S. Ayotte B. Edelstein B. Margrett J. (2008). Framing effect debiasing in medical decision making. Patient Educ. Couns. 71, 102–107. 10.1016/j.pec.2007.11.004 18164168

[B3] Arendell T. (2000). Conceiving and investigating motherhood: The decade's scholarship. J. Marriage Fam. 62, 1192–1207. 10.1111/j.1741-3737.2000.01192.x

[B4] Arora N. K. McHorney C. A. (2000). Patient Preferences for medical decision making: who really wants to participate? Med. Care 38, 335–341. 10.1097/00005650-200003000-00010 10718358

[B5] Baron J. Hershey J. C. (1988). Outcome bias in decision evaluation. J. Pers. Soc. Psychol. 54, 569–579. 10.1037/0022-3514.54.4.569 3367280

[B6] Baron J. Scott S. Fincher K. Metz S. E. (2015). Why does the cognitive reflection test (Sometimes) predict utilitarian moral judgment (and other things)? J. Appl. Res. Mem. Cogn. 4, 265–84. 10.1016/j.jarmac.2014.09.003

[B7] Bedford H. (2014). Pro-vaccine messages may be counterproductive among vaccine-hesitant parents. Evid. Based Med. 19:219. 10.1136/ebmed-2014-110037 25185270

[B8] Berg-Cross L. G. (1975). Intentionality, degree of damage, and moral judgments. Child Dev. 46, 970–974. 10.2307/1128406 1201671

[B9] Betsch C. Renkewitz F. Betsch T. Ulshöfer C. (2010). The influence of vaccine-critical websites on perceiving vaccination risks. J. Health Psychol. 15, 446–455. 10.1177/1359105309353647 20348365

[B10] Bloom B. R. Marcuse E. Mnookin S. (2014). Addressing vaccine hesitancy. Science 344, 339. 10.1126/science.1254834 24763557

[B11] Boes L. Boedeker B. Schmich P. Wetzstein M. Wichmann O. Remschmidt C. (2017). Factors associated with parental acceptance of seasonal influenza vaccination for their children–A telephone survey in the adult population in Germany. Vaccine 30, 3789–3796. 10.1016/j.vaccine.2017.05.015 28558985

[B12] Brunson E. K. (2013a). How parents make decisions about their children's vaccinations. Vaccine 31, 5466–5470. 10.1016/j.vaccine.2013.08.104. 24076175

[B13] Brunson E. K. (2013b). The impact of social networks on parents' vaccination decisions. Pediatrics 131, 1397–1404. 10.1542/peds.2012-2452 23589813

[B14] Callender D. (2016). Vaccine hesitancy: more than a movement. Hum. Vaccin. Immunother. 12, 2464–2468. 10.1080/21645515.2016.1178434 27159558 PMC5027704

[B15] Camargo K. Jr. Grant R. (2015). Public health, science, and policy debate: being right is not enough. Am. J. Public Health 105, 232–235. 10.2105/AJPH.2014.302241 25521880 PMC4318315

[B16] Carrico A. R. Vandenbergh M. P. Stern P. C. Gardner G. T. Dietz T. Gilligan J. M. (2011). Energy and climate change: key lessons for implementing the behavioral wedge. George Washington J. Ener. Environ. Law 2, 10–24. Available online at: https://ssrn.com/abstract=1612224

[B17] Cobos M. D. Monzón Llamas L. Bosch-Capblanch X. (2015). Exposing concerns about vaccination in low- and middle-income countries: a systematic review. Int. J. Public Health 60, 767–780. 10.1007/s00038-015-0715-6 26298444

[B18] Collins H. (2009). We Cannot Live by Scepticism Alone. Nature 458:30 10.1038/458030a 19262652

[B19] Contractor S. H. Fox R. J. (2011). An investigation of the relationship between the number of response categories and scale sensitivity. J. Targe. Meas. Anal. Market. 19, 23–35. 10.1057/jt.2011.4

[B20] Damnjanović K. Gvozdenović V. (2016). Influence of the probability level on the framing effect. Psihologijske Teme 25, 405–429. Available online at: https://hrcak.srce.hr/169520

[B21] Darwin H. Neave N. Holmes J. (2011). Belief in conspiracy theories. the role of paranormal belief, paranoid ideation and schizotypy. Pers. Individ. Diff. 50, 1289–1293. 10.1016/j.paid.2011.02.027

[B22] de Visser R. Waites L. Parikh C. Lawrie A. (2011). The importance of social norms for uptake of catch-up human papillomavirus vaccination in young women. Sex. Health 8, 330–337. 10.1071/SH10155 21851772

[B23] Donovan R. J. Jalleh G. (2000). Positive versus negative framing of a hypothetical infant immunization: the influence of involvement. Health Educ. Behav. 27, 82–95. 10.1177/109019810002700108 10709794

[B24] Downs J. S. de Bruin W. B. Fischhoff B. (2008). Parents' vaccination comprehension and decisions. Vaccine 26, 1595–1607. 10.1016/j.vaccine.2008.01.011 18295940

[B25] Dubé E. Gagnon D. Nickels E. Jeram S. Schuster M. (2014). Mapping vaccine hesitancy-Country-specific characteristics of a global phenomenon. Vaccine 32, 6649–6654. 10.1016/j.vaccine.2014.09.039 25280436 PMC5355208

[B26] Edwards A. Elwyn G. Covey J. Matthews E. Pill R. (2001). Presenting risk information a review of the effects of framing and other manipulations on patient outcomes. J. Health Commun. 6, 61–82. 10.1080/10810730150501413 11317424

[B27] Ember C. R. Ember M. Peregrine N. (1998). Cross-cultural research, in Handbook of Methods in Cultural Anthropology, ed Bernard H. R. (Walnut Creek, CA: AltaMira), 647–687.

[B28] Eurostat (2017). Inability To Make Ends Meet – Eurostat. Ec.Europa.Eu. Available online at: http://ec.europa.eu/eurostat/en/web/products-datasets/-/ILC_MDES09

[B29] Evans M. Stoddart H. Condon L. J. Freeman E. Grizzell M. Mullen R. (2001). Parents' perspectives on the MMR immunisation: a focus group study. Br. J. Gen. Pract. 51, 904–910. Available online at: http://bjgp.org/content/bjgp/51/472/904.full.pdf 11761204 PMC1314147

[B30] Fagley N. S. Miller P. M. (1997). Framing effects and Arenas of choice: your money or your life? Org. Behav. Hum. Decis. Proc. 71, 355–373. 10.1006/obhd.1997.2725

[B31] Freed G. L. Clark S. J. Butchart A. T. Singer D. C. Davis M. M. (2010). Parental vaccine safety concerns in 2009. Pediatrics 125, 654–659. 10.1542/peds.2009-1962 20194286

[B32] Freed G. L. Clark S. J. Butchart A. T. Singer D. C. Davis M. M. (2011). Sources and perceived credibility of vaccine-safety information for parents. Pediatrics 127(Suppl. 1), S107–S112. 10.1542/peds.2010-1722P 21502236

[B33] Freed G. L. Clark S. J. Hibbs B. F. Santoli J. M. (2004). Parental vaccine safety concerns: the experiences of pediatricians and family physicians. Am. J. Prevent. Med. 26, 11–14. 10.1016/j.amepre.2003.09.004 14700706

[B34] GBD 2015 Healthcare Access and Quality Collaborators (2017). Healthcare access and quality index based on mortality from causes amenable to personal health care in 195 countries and territories. a novel analysis from the global burden of disease study 2015. Lancet 390, 231–266. 10.1016/S0140-6736(17)30818-8 28528753 PMC5528124

[B35] Gellin B. G. Maibach E. W. Marcuse E. K. (2000). Do parents understand immunizations? A national telephone survey. Pediatrics 106, 1097–1102. 10.1542/peds.106.5.1097 11061781

[B36] Gino F. Shu L. Bazerman M. (2010). Nameless + harmless = blameless: when seemingly irrelevant factors influence judgment of (un)ethical behavior. Organ. Behav. Hum. Decis. Process. 111, 93–101. 10.1016/j.obhdp.2009.11.001

[B37] Goldenberg M. J. (2016). *Public Misunderstanding of Science*? Reframing the Problem of Vaccine Hesitancy. Perspectives on Science. Cambridge, MA: MIT Press.

[B38] Goldstein W. M. Weber E. U. (1995). Content and discontent: indications and implications of domain specificity in preferential decision making. Psychol. Learn. Motivat. 32, 83–136.

[B39] Gowda C. Dempsey A. F. (2013). The rise (and Fall?) of parental vaccine hesitancy. Hum. Vacc. Immunother. 9, 1755–1762. 10.4161/hv.25085 23744504 PMC3906278

[B40] Gowda C. Carlos R. C. Butchart A. T. Singer D. C. Davis M. M. Dempsey A. F. (2012). CHIAS: a standardized measure of parental HPV immunization attitudes and beliefs and its associations with vaccine uptake. Sex. Transm. Dis. 39, 475–481. 10.1097/OLQ.0b013e318248a6d5 22592835

[B41] Grol R. Whitfield M. De Maeseneer J. Mokkink H. (1990). Attitudes to risk taking in medical decision making among British, Dutch and Belgian general practitioners. Br. J. Gen. Pract. 40, 134–136. 2115347 PMC1371238

[B42] Gummerum M. Hanoch Y. Rolison J. J. (2014). Offenders' risk-taking attitude inside and outside the prison walls Risk Anal. 34, 1870–1881. 10.1111/risa.12222 24913147

[B43] Gust D. A. Darling N. Kennedy A. Schwartz B. (2008). Parents with doubts about vaccines: which vaccines and reasons why. Pediatrics 122, 718–725. 10.1542/peds.2007-0538 18829793

[B44] Gust D. A. Kennedy A. Shui I. Smith P. J. Nowak G. Pickering L. K. (2005). Parent attitudes toward immunizations and healthcare providers: the role of information. Am. J. Prev. Med. 29, 105–112. 10.1016/j.amepre.2005.04.010 16005806

[B45] Gust D. A. Strine T. W. Maurice E. Smith P. Yusuf H. Wilkinson M.. (2004). Underimmunization among children: effects of vaccine safety concerns on immunization status. Pediatrics 114, 16–21. 10.1542/peds.114.1.e16 15231968

[B46] Hanoch Y. Johnson J. G. Wilke A. (2006). Domain specificity in experimental measures and participant recruitment: an application to risk-taking behavior. Psychol. Sci. 17, 300–304. 10.1111/j.1467-9280.2006.01702.x 16623686

[B47] Haran U. Ritov I. Mellers B. A. (2013). The role of actively open-minded thinking in information acquisition, accuracy, and calibration. Judgm. Decis. Mak. 8, 188–201. 10.1177/2053168016676705

[B48] Hays S. (1996). The Cultural Contradictions of Motherhood. New Haven, CT: Yale University Press.

[B49] Healy C. M. Pickering L. K. (2011). How to communicate with vaccine-hesitant parents. Pediatrics 127 (Suppl. 1), S127–S133. 10.1542/peds.2010-1722S 21502238

[B50] Henrikson N. B. Anderson M. L. Opel D. J. Dunn J. Marcuse E. K. Grossman D. C. (2017). Longitudinal Trends in Vaccine Hesitancy in a Cohort of Mothers Surveyed in Washington State, 2013-2015. Public Health Reports. SAGE Publications, Sage, CA, 451–454. 10.1177/0033354917711175 PMC5507431 28586623

[B51] Highland J. (2016). Parental Decision Making and Childhood Vaccination. CaseWestern Reserve University, 1–30.

[B52] Hobson-West P. (2007). Trusting Blindly Can Be the Biggest Risk of All': organised resistance to childhood vaccination in the UK. Sociol. Health Illness 29, 198–215. 10.1111/j.1467-9566.2007.00544.x 17381813

[B53] Horne Z. Powell D. Hummel J. E. Holyoak K. J. (2015). Countering antivaccination attitudes. Proc. Natl. Acad. Sci. U.S.A. 112, 10321–10324. 10.1073/pnas.1504019112 26240325 PMC4547299

[B54] Iyengar S. S. Lepper M. R. (2000). When choice is demotivating: can one desire too much of a good thing? J. Pers. Soc. Psychol. 79, 995–1006. 10.1037/0022-3514.79.6.995 11138768

[B55] Jolley D. Douglas K. M. (2014). The effects of anti-vaccine conspiracy theories on vaccination intentions. PLoS ONE 9:e89177. 10.1371/journal.pone.0089177 24586574 PMC3930676

[B56] Jones A. M. Omer S. B. Bednarczyk R. A. Halsey N. A. Moulton L. H. Salmon D. A. (2012). Parents' source of vaccine information and impact on vaccine attitudes, beliefs, and nonmedical exemptions. Adv. Prevent. Med. 2012, 1–9. 10.1155/2012/932741 23082253 PMC3469070

[B57] Kahan D. M. (2010). Fixing the communications failure. Nature 463, 296–297. 10.1038/463296a 20090734

[B58] Kahan D. M. Braman D. (2006). Cultural cognition and public policy. Yale Law Policy Rev. 24, 147–170. Available online at: http://digitalcommons.law.yale.edu/ylpr/vol24/iss1/5

[B59] Kahan D. M. Braman D. Cohen G. L. Gastil J. Slovic P. (2010). Who fears the HPV vaccine, who doesn't, and why? an experimental study of the mechanisms of cultural cognition. Law Hum. Behav. 34, 501–516. 10.1007/s10979-009-9201-0 20076997

[B60] Kahan D. M. Braman D. Slovic P. Gastil J. Cohen G. L. (2009). Cultural cognition of the risks and benefits of nanotechnology. Nat. Nanotechnol. 4, 87–90. 10.1038/nnano.2008.341 19197308

[B61] Kahan D. M. Jenkins-Smith H. Braman D. (2011). Cultural cognition of scientific consensus. J. Risk Res. 14, 147–174. 10.1080/13669877.2010.511246

[B62] Keinan R. Bereby-Meyer Y. (2012). ‘Leaving It to chance’ —passive risk taking in everyday life. Judgm. Decis. Mak. 7, 705–715. Available online at: http://journal.sjdm.org/15/15717/jdm15717.pdf

[B63] Kennedy A. LaVail K. Nowak G. Basket M. Landry S. (2011). Confidence about vaccines in the United States: understanding parents' perceptions. Health Aff. 30, 1151–1159. 10.1377/hlthaff.2011.0396 21653969

[B64] Kühberger A. (1998). The influence of framing on risky decisions: a meta-analysis. Organ. Behav. Hum. Decis. Process. 75, 23–55. 10.1006/obhd.1998.2781 9719656

[B65] Larson H. J. Jarrett C. Eckersberger E. Smith D. M. D. Paterson P. (2014). Understanding vaccine hesitancy around vaccines and vaccination from a global perspective: a systematic review of published literature, 2007–2012. Vaccine 32, 2150–2159. 10.1016/j.vaccine.2014.01.081 24598724

[B66] Lau S. Hiemisch A. Baumeister R. F. (2015). The experience of freedom in decisions—questioning philosophical beliefs in favor of psychological determinants. Conscious. Cogn. 33, 30–46. 10.1016/j.concog.2014.11.008 25528494

[B67] Leask J. A. Braunack-Mayer A. Kerridge I. (2011). Consent and public engagement in an era of expanded childhood immunisation. J. Paediatr. Child Health 47, 603–607. 10.1111/j.1440-1754.2011.02160.x 21951441

[B68] Leask J. MacArtney K. (2008). Parental decisions about vaccination: collective values are important. J. Paediatr. Child Health 44, 534–535. 10.1111/j.1440-1754.2008.01381.x 19012625

[B69] Lewandowsky S. Oberauer K. (2016). Motivated rejection of science. Curr. Dir. Psychol. Sci. 25, 217–222. 10.1177/0963721416654436

[B70] Lewandowsky S. Ecker U. K. H. Seifert C. M. Schwarz N. Cook J. (2012). Misinformation and its correction. Psychol. Sci. Pub. Interest 13, 106–131. 10.1177/1529100612451018 26173286

[B71] Lipshitz R. (1989). ‘Either a Medal or a Corporal’: the effects of success and failure on the evaluation of decision making and decision makers. Organ. Behav. Hum. Decis. Process. 44, 380–395.

[B72] Luthy K. E. Beckstrand R. L. Peterson N. E. (2009). Parental hesitation as a factor in delayed childhood immunization. J. Pediatr. Health Care 23, 388–393. 10.1016/j.pedhc.2008.09.006 19875026

[B73] Markiewicz Ł. Weber E. U. (2013). DOSPERT's gambling risk-taking propensity scale predicts excessive stock trading. J. Behav. Finance 14, 65–78. 10.1080/15427560.2013.762000

[B74] McNeil B. J. Pauker S. G. Sox H. C. Jr. Tversky A. (1982). On the elicitation of preferences for alternative therapies. N. Engl. J. Med. 306, 1259–1262. 10.1056/NEJM198205273062103 7070445

[B75] Mills E. J. Montori V. M. Ross C. P. Shea B. Wilson K. Guyatt G. H. (2005). Systematically reviewing qualitative studies complements survey design: an exploratory study of barriers to paediatric immunisations. J. Clin. Epidemiol. 58, 1101–1108. 10.1016/j.jclinepi.2005.01.014 16223652

[B76] Miton H. Mercier H. (2015). Cognitive obstacles to pro-vaccination beliefs. Trends Cogn. Sci. 19, 633–636. 10.1016/j.tics.2015.08.007 26522341

[B77] Mohd Azizi F. S. Kew Y. Moy F. M. (2017). Vaccine hesitancy among parents in a multi-ethnic country, Malaysia. Vaccine 35, 2955–2961. 10.1016/j.vaccine.2017.04.010 28434687

[B78] Myers L. B. Goodwin R. (2011). Determinants of adults' intention to vaccinate against pandemic swine flu. BMC Public Health 11:15. 10.1186/1471-2458-11-15 21211000 PMC3024930

[B79] Nenkov G. Y. Inman J. J. Hulland J. (2008). Considering the future: the conceptualization. J. Consu. Res. 35, 126–141. 10.1086/525504

[B80] Opel D. J. Taylor J. A. Mangione-Smith R. Solomon C. Zhao C. Martin D.. (2011). Validity and reliability of a survey to identify vaccine-hesitant parents. Vaccine 29, 6598–6605. 10.1016/j.vaccine.2011.06.115 21763384

[B81] Oraby T. Thampi V. Bauch C. T. (2014). The influence of social norms on the dynamics of vaccinating behaviour for paediatric infectious diseases. Proc. Biol. Sic. 281:20133172. 10.1098/rspb.2013.3172 24523276 PMC4078885

[B82] Pareek M. Pattison H. M. (2000). The two-dose Measles, Mumps, and Rubella (MMR) immunisation schedule: factors affecting maternal intention to vaccinate. Br. J. Gen. Pract. 50, 969–971. 11224968 PMC1313883

[B83] Payne J. W. (1982). Contingent decision behavior. Psychol. Bull. 92, 382–402. 10.21236/ADA111655

[B84] Payne J. W. Bettman J. R. Johnson E. J. (1988). Adaptive strategy selection in decision making. J. Exp. Psychol. Learn. Mem. Cogn. 14, 534–552. 10.1037/0278-7393.14.3.534

[B85] Payne J. W. Bettman J. R. Johnson E. J. (1993). The Adaptive Decision Maker.

[B86] Pearce A. Law C. Elliman D. Cole T. J. Bedford H. (2008). Factors associated with uptake of measles, mumps, and rubella vaccine (MMR) and use of single antigen vaccines in a contemporary UK cohort: prospective cohort study. BMJ 336, 754–757. 10.1136/bmj.39489.590671.25 18309964 PMC2287222

[B87] Pluviano S. Watt C. Sala S. D. (2017). Misinformation lingers in memory: failure of three pro-vaccination strategies. PLoS ONE 12:e0181640. 10.1371/journal.pone.0181640 28749996 PMC5547702

[B88] Pyke-Grimm K. A. Degner L. Small A. Mueller B. (1999). Preferences for participation in treatment decision making and information needs of parents of children with cancer: a pilot study. J. Pediatr. Oncol. Nurs. 16, 13–24. 9989013 10.1177/104345429901600103

[B89] Rachiotis G. Mouchtouri V. A. Kremastinou J. Gourgoulianis K. Hadjichristodoulou C. (2010). Low Acceptance of Vaccination against the 2009 Pandemic Influenza A (H1N1) among Healthcare Workers in Greece. Euro Surveill. 15:19486. 10.2807/ese.15.06.19486-en 20158980

[B90] Reich J. A. (2014). Neoliberal mothering and vaccine refusal: imagined gated communities and the privilege of choice. Gender Soc. 28, 679–704. 10.1177/0891243214532711

[B91] Reyna V. F. (2008). A theory of medical decision making and health: fuzzy trace theory. Med. Decis. Making 28, 850–865. 10.1177/0272989X08327066 19015287 PMC2617718

[B92] Reyna V. F. Brainerd C. J. (1992). A fuzzy-trace theory of reasoning and remembering: paradoxes, patterns, and parallelism, in From Learning Processes to Cognitive Processes: Essays in Honor of William K. Estes 2, eds Healy A. F. Kosslyn S. M. Shiffrin R. M. (Hillsdale, NJ: Erlbaum), 235–259.

[B93] Rolfe-Redding J. C. Maibach E. W. Feldman L. Leiserowitz A. (2012). Republicans and climate change: an audience analysis of predictors for belief and policy preferences, in 62nd Annual International Communication Association Conference (Phoenix, AZ), 1–48.

[B94] Ruiter R. A. C. Abraham C. Kok G. (2001). Scary warnings and rational precautions: a review of the psychology of fear appeals. Psychol. Health 16, 613–630. 10.1080/08870440108405863

[B95] Ruiter R. A. Kessels L. T. Peters G. J. Kok G. (2014). Sixty years of fear appeal research: current state of the evidence. Int. J. Psychol. 49, 63−70. 10.1002/ijop.12042 24811876

[B96] Sadaf A. Richards J. L. Glanz J. Salmon D. A. Omer S. B. (2013). A systematic review of interventions for reducing parental vaccine refusal and vaccine hesitancy. Vaccine 31, 4293–4304. 10.1016/j.vaccine.2013.07.013 23859839

[B97] Salmon D. A. Moulton L. H. Omer S. B. M. Patricia deHart Stokley S. Halsey N. A. (2005). Factors associated with refusal of childhood vaccines among parents of school-aged children: a case-control study. Arch. Pediatrics Adoles. Med. 159, 470–476. 10.1001/archpedi.159.5.470 15867122

[B98] Seeman N. Ing A. Rizo C. (2010). Assessing and responding in real time to online anti-vaccine sentiment during a flu pandemic. Healthcare Q. 13, 8–15. 10.12927/hcq.2010.21923 20959725

[B99] Shapiro G. K. Holding A. Perez S. Amsel R. Rosberger Z. (2016). Validation of the vaccine conspiracy beliefs scale. Papillomavirus Res. 2, 167–172. 10.1016/j.pvr.2016.09.001 29074176 PMC5886898

[B100] Smailbegovic M. S. Laing G. J. Bedford H. (2003). Why do parents decide against immunization? the effect of health beliefs and health professionals. Child Care Health Dev. 29, 303–311. 10.1046/j.1365-2214.2003.00347.x 12823336

[B101] Smith P. J. Susan Chu Y. Lawrence Barker E. (2004). Children who have received no vaccines: who are they and where do they live? Pediatrics 114, 187–195. 10.1542/peds.114.1.187 15231927

[B102] Smyth C. Craig L. (2017). Conforming to intensive parenting ideals: willingness, reluctance and social context. Famil. Relationsh. Soc. 6, 107–124. 10.1332/204674315X14393034138937

[B103] Solomon M. G. Bamossy S. Askegaard Hogg M. K. (2006). Consumer Behaviour: A European Perspective, 3rd Edn. New Jersey, NJ: Prentice Hall Inc.

[B104] Sporton R. K. Francis S. A. (2001). Choosing not to immunize: are parents making informed decisions? Fam. Pract. 18, 181–188. 10.1093/fampra/18.2.181 11264269

[B105] Stanovich K. E. West R. F. (2007). Natural myside bias is independent of cognitive ability. Think. Reason. 13, 225–247. 10.1080/13546780600780796

[B106] Stojkovic A. Lazarevic J. Anzelm D. Drljaca U. Zezelj I. Damnjanovic K. (2017). Psychological Correlates of Resistance to Mandatory Child Vaccination. University of Zadar Department of Psychology, ZADAR, CROATIA.

[B107] Stronach A. E. (2015). Media and Internet Influences on Parental Decision Making Related to Vaccination. Christchurch: University of Canterbury.

[B108] Swami V. Coles R. Stieger S. Pietschnig J. Furnham A. Rehim S.. (2011). Conspiracist ideation in britain and austria: evidence of a monological belief system and associations between individual psychological differences and real-world and fictitious conspiracy theories. Br. J. Psychol. 102, 443–463. 10.1111/j.2044-8295.2010.02004.x 21751999

[B109] Tanner C. Medin D. L. Iliev R. (2008). Influence of deontological versus consequentialist orientations on act choices and framing effects: when principles are more important than consequences. Eur. J. Soc. Psychol. 38, 757–69. 10.1002/ejsp.493

[B110] Thompson A. G. H. (2007). The meaning of patient involvement and participation in health care consultations: a taxonomy. Soc. Sci. Med. 64, 1297–1310. 10.1016/j.socscimed.2006.11.002 17174016

[B111] Timmermans D. (1993). The impact of task complexity on information use in multi-attribute decision making. J. Behav. Decis. Making 6, 95–111.

[B112] Tom J. (2017). Social origins of scientific deviance: examining creationism and global warming skepticism. Sociol. Perspect. 61, 341–360. 10.1177/0731121417710459

[B113] Van der Linden S. (2011). Charitable intent: a moral or social construct? a revised theory of planned behavior model. Curr. Psychol. 30, 355–374. 10.1007/s12144-011-9122-1

[B114] van der Linden S. L. Leiserowitz A. Rosenthal S. Maibach E. (2017). Inoculating the public against misinformation about climate change. Global Challenges 1:1600008 10.1002/gch2.201600008 PMC6607159 31565263

[B115] Van der Linden S. L. Maibach E. Leiserowitz A. (2015). Improving public engagement with climate change. Perspect. Psychol. Sci. 10, 758–763. 10.1177/1745691615598516 26581732

[B116] Van der Linden S. Lewandowsky S. (2015). How to combat distrust of science: the surprising power of the psychology of consensus. Sci. Am. Mind. Available online at: https://www.scientificamerican.com/article/how-to-combat-distrust-of-science/ (Accessed October 20, 2017).

[B117] Wagenaar W. A. Keren G. Lichtenstein S. (1988). Islanders and hostages: deep and surface structures of decision problems. Acta Psychol. 67, 175–189. 10.1016/0001-6918(88)90012-1

[B118] Wang X. T. (1996a). Framing effects: dynamics and task domains. organizational behavior and human decision processes. Organ. Behav. Human Decis. Process. 68, 145–157. 10.1006/obhd.1996.0095 8954876

[B119] Wang X. T. (1996b). Domain-specific rationality in human choices: violations of utility axioms and social contexts. Cognition 60, 31–63. 8766389 10.1016/0010-0277(95)00700-8

[B120] Ward P. R. Attwell K. Meyer S. B. Rokkas P. Leask J. (2017). Understanding the perceived logic of care by vaccine-hesitant and vaccine-refusing parents: a qualitative study in Australia. PLoS ONE 12:e0185955. 10.1371/journal.pone.0185955 29023499 PMC5638294

[B121] Wilson R. S. Arvai J. L. Arkes H. R. (2008). My loss is your loss. sometimes: loss aversion and the effect of motivational biases. Risk Anal. 28, 929–938. 10.1111/j.1539-6924.2008.01065.x 18564994

[B122] World Health Organization (2014). Report of the SAGE Working Group on Vaccine Hesitancy. Available online at: http://www.who.int/immunization/sage/meetings/2014/october/1_Report_WORKING_GROUP_vaccine_hesitancy_final.pdf (Accessed October 28, 2017).

[B123] World Health Organization (2017a). Density of Physicians (Total Number per 1000 Population, Latest Available Year). Available online at: http://www.who.int/gho/health_workforce/physicians_density/en/

[B124] World Health Organization (2017b). Immunization Covered. Available online at: http://www.who.int/mediacentre/factsheets/fs378/en/

[B125] World Health Organization (2018). Europe Observes a 4 Fold Increase in Measles Cases in 2017 Compared to a Previous Year. Available online at: http://www.euro.who.int/en/media-centre/sections/press-releases/2018/europe-observes-a-4-fold-increase-in-measles-cases-in-2017-compared-to-previous-year

[B126] Wright P. (1975). Consumer choice strategies: simplifying vs. optimizing. J. Market. Res. 12, 60–67. 10.2307/3150659

[B127] Wroe A. L. Turner N. Salkovskis P. M. (2004). Understanding and predicting parental decisions about early childhood immunizations. Health Psychol. 23, 33–44. 10.1037/0278-6133.23.1.33 14756601

[B128] Ziegler G. Durus N. Sert O. Family N. (2015). Analysing ELT in the european arena: multilingual practices, in International Perspectives on ELT Classroom Interaction, eds Jenks C. J. Seedhouse P. (London, UK: Palgrave), 188–207.

[B129] Zikmund-Fisher B. J. Sarr B. Fagerlin A. Ubel P. A. (2006). A matter of perspective: choosing for others differs from choosing for yourself in making treatment decisions. J. Gener. Int. Med. 21, 618–22. 10.1111/j.1525-1497.2006.00410.x 16808746 PMC1924622

[B130] Zimerman L. Shalvi S. Bereby-Meyer Y. (2014). Self-reported ethical risk taking tendencies predict actual dishonesty. Judgm. Decis. Mak. 9, 58–64. Available online at: http://journal.sjdm.org/13/131111/jdm131111.pdf

[B131] Zingg A. Siegrist M. (2012). Measuring people's knowledge about vaccination: developing a one-dimensional scale. Vaccine 30, 3771–3777. 10.1016/j.vaccine.2012.03.01 22445808

