# Supplementary material for: Parental Decision-Making on Childhood Vaccination
Source: Front Psychol. 2018 Jun 13;9:735. doi: 10.3389/fpsyg.2018.00735 (PMC6008886; doi:10.3389/fpsyg.2018.00735)
Supplement: Supplementary file 1 [file Data_Sheet_1.pdf]

Supplementary Material

Parental Decision-Making in Childhood Vaccination

Kaja Damnjanović*, Johanna Graeber, Sandra Ilić, Wing Yi Lam, Žan Lep*, Sara Morales, Tero Pulkkinen and Loes Vingerhoets

*** Correspondence:** Kaja Damnjanović: kdamnjan@f.bg.ac.rs
Žan Lep: zan.lep@empirik.si

Complete battery for Study 1

1 Demographics

Country of residence
Please select your gender:

a) Woman
b) Man
c) Other

Age *(in arabic numbers)*
Please select the highest education level that you completed *(options adapted for each country)*
Please, select your marital status:

a) Single
b) Married
c) Divorced or separated

Please select the option that best fits your actual situation:

a) Living with the other parent of the child/ children
b) Living with another partner (not the parent)
c) Single parent

To what extent do you find difficulties to make the ends meet in your household?
Likert scale (1=Not difficult at all to 7=completely difficult)

How many children do you have or are currently taking care of?

How old are they?

a) Age of the child 1:
b) Age of the child 2:
c) Age of the child 3:
d) Age of the child 4:
e) Age of the child 5:
f) Age of the child 6:

g) Age of the child 7:

Are the children vaccinated according to the official vaccination schedule in your country

a) Yes, all vaccines in the schedule have been administered at the recommended age.
b) Some of the vaccines have not been administered following the official schedule
c) No, the children are not being vaccinated at all
d) Other possibilities (open-ended)

If you selected the second or third option, please briefly state the reason(s) why your children are not being vaccinated according to the official schedule of your county (open-ended)

Have **you** been vaccinated according to the official vaccination schedule (at the time) in your country?

a) Yes, all vaccines in the schedule have been administered at the recommended age.
b) Some of the vaccines have not been administered following the official schedule
c) No, I have not been vaccinated at all
d) Other possibilities (open-ended)

If you selected the second or third option, please briefly state the reason(s) why you have not been vaccinated according to the official schedule of your county (open-ended)

2 Dependant variable

Would you at this time vaccinate your child according to the official vaccination schedule?
*Likert 7-point scale (Definitely not=1****;*** *Definitely yes=7)*

3 Psychological features

3.1 Vaccine hesitancy

Please, rate your agreement to each of this items in the following scale, where 1 means “I strongly disagree”, and 7 means “I strongly agree”:

1. The risk of side effects outweighs any protective benefits of vaccines.
2. Vaccinating healthy children helps protect others by stopping the spread of disease.
3. I plan to vaccinate my children.
4. Children do not need vaccines for diseases that are not common anymore.
5. Doctors would not recommend vaccines if they were unsafe
6. Vaccine safety data is often fabricated.
7. Immunizing children is harmful and this fact is covered up.
8. Pharmaceutical companies cover up the dangers of vaccines.
9. People are deceived about vaccine efficacy.
10. Vaccine efficacy data is often fabricated.
11. The government is trying to cover up the link between vaccines and autism.

Please, complete the following survey:

1. Have you ever delayed having your child get a shot for reasons other than illness or allergy?
   a) Yes
   b) No
   c) I don’t know

2. Have you ever decided not to have your child get a shot for reasons other than illness or allergy?
    a) Yes
    b) No
    c) I don't know
3. If you had another infant today, would you want him/her to get all the recommended shots?
    a) Yes
    b) No
    c) I don't know
4. How sure are you that following the recommended shot schedule is a good idea for your child?
    Likert 7-point scale (1=Not sure at all; 7= Completely sure)
5. Children get more shots than are good for them
    Likert 7-point scale (1=Completely disagree; 7=Completely agree)
6. I believe that many of the illnesses shots prevent are severe
    Likert 7-point scale (1=Completely disagree; 7=Completely agree)
7. It is better for my child to develop immunity by getting sick than to get a shot.
    Likert 7-point scale (1=Completely disagree; 7=Completely agree)
8. It is better for children to get fewer vaccines at the same time.
    Likert 7-point scale (1=Completely disagree; 7=Completely agree)
9. How concerned are you that your child might have a serious side effect from a shot?
    Likert 7-point scale (1=Not concerned at all; 7=Completely concerned)
10. How concerned are you that any one of the childhood shots might not be safe?
    Likert 7-point scale (1=Not concerned at all; 7=Completely concerned)
11. How concerned are you that a shot might not prevent the disease?
    Likert 7-point scale (1=Not concerned at all; 7=Completely concerned)
12. Overall, how hesitant about childhood shots would you consider yourself to be?
    Likert 7-point scale (1=Not concerned at all; 7=Completely concerned)
13. I trust the information I receive about shots.
    Likert 7-point scale (1=Completely disagree; 7=Completely agree)
14. I am able to openly discuss my concerns about shots with my child's doctor.
    Likert 7-point scale (1=Completely disagree; 7=Completely agree)

3.2 Perceived freedom

When I was making the decision of whether to vaccinate my child/children…

1. I felt very free in this decision
2. I was able to choose what I wanted
3. I was able to ponder and think freely and easily
4. I felt very constricted in this decision

Likert 7-point scale (Definitely not=1; Definitely yes=7)

3.3 Choice overload

When I was making the decision of whether to vaccinate my child/children…

1. I felt overwhelmed by the decision
2. The choice exhausted me
3. It was difficult to keep all the relevant information together

Likert 7-point scale (Definitely no=1; Definitely yes=7)

3.4 The Actively Open-Minded Thinking Scale

Please, rate to what extent do you agree with these statements.

1. Allowing oneself to be convinced by an opposing argument is a sign of good character.
2. People should take into consideration evidence that goes against their beliefs.
3. People should revise their beliefs in response to new information or evidence.
4. Changing your mind is a sign of weakness.
5. Intuition is the best guide in making decisions.
6. It is important to persevere in your beliefs even when evidence is brought to bear against them.
7. One should disregard evidence that conflicts with one's established beliefs.
8. People should search actively for reasons why their beliefs might be wrong.
9. When we are faced with a new question, the first answer that occurs to us is usually best.
10. When faced with a new question, we should consider more than one possible answer before reaching a conclusion.
11. When faced with a new question, we should look for reasons why our first answer might be wrong, before deciding on an answer.

Likert 7-point scale (Completely disagree=1; Completely agree=7)

3.5 Trust towards authorities

Please indicate the extent to which you trust the following sources to tell the truth about vaccinations:

a) Corporations
b) National government
c) Healthcare system
d) Science/scientist
e) Mainstream media
f) Alternative media
g) Social networks
h) Your child's doctor

Likert 7-point scale (Completely mistrust=1; Completely trust=7)

3.6 Sources of information

Please check all the sources you have used to make the decision about vaccination

a) Social Networks
b) Mainstream media
c) Alternative media
d) Government communication
e) Friends and family
f) Books
g) Magazines
h) Scientific journals
i) My children's paediatrician
j) Websites concerning vaccination
k) Others: ____________

3.7 Access to information

I have access to all the information I need to make good decisions about immunizing my children.
Likert 7-point scale (Definitely no=1; Definitely yes=7)

3.8 Exposure to anecdotal cases

Have you ever heard of anyone who has had a bad reaction to a shot?

a) Yes
b) No

3.9 Involvement in the decision

Please, rate your level of involvement in the decision of vaccinating your children or not
Likert 7-point scale (Not involved at all =1; Completely involved=7)
Was anyone else also involved in the decision?

a) Yes
    i. How many people were also involved in the decision?
    ii. Who did you consult?
        1. The other parent of the kid
        2. Another family member
        3. A friend
        4. Another person
    iii. Gender(s)
        1. Male
        2. Female
        3. Other
    iv. Please rate the person(s)'s level of involvement in the decision.
    Likert 7-point scale (Not involved at all =1; Completely involved=7)
b) No

3.10 Norms, perceived consensus, knowledge

Is there a consensus among scientists about the safety of vaccines?
Likert 7-point scale (No consensus at all=1; Total consensus=7)
Is there a consensus among scientists about the effectiveness of vaccines?
Likert 7-point scale (No consensus at all=1; Total consensus=7)
What do you think is the percentage of vaccinated children in your country?
0% to 100% scale
Is vaccination mandatory in your country?

a) Yes
b) No

Is the "vaccination issue" a big thing in your country?
Likert 7-point scale (Definitely no=1; Definitely yes=7)

3.11 Passive Risk Taking

To what extent are you likely to behave in the manner described in the following situations?

1. Buy an expensive product (computer, refrigerator) only after comparing prices in several stores.
2. Install an up to date anti -virus on my computer.
3. Check the credit card bill in detail every month.

4. Inquire all about a course before signing up (who is the lecturer, what are the topics, the assignments etc.…
5. Read the fine print on any major document like a lease, an insurance policy or loan
6. Save receipts and warranty documents of major items in an organized fashion.
7. Check tolls and prices before calling long distance or overseas.
8. Back up all important files on the computer, including documents, pictures or videos.
9. Not save money regularly
10. Always lock the house door when going to sleep
11. Buy clothes without trying them on
12. Buy a used car only after taking it to a complete check up in a licensed auto shop
13. Immediately go to the doctor's when something in my body is aching or bothering me.
14. Have regular general medical check -ups every one or two years.
15. Get vaccinated for the flu in the winter.
16. Install an anti -collision device in the car.
17. Drive straight to the auto repair shop when the car makes a strange noise.
18. Ask the person I am dating about his/her sexual history.
19. Buy serious medical insurance when traveling to another country.
20. Always wear a seatbelt when sitting in the back seat.
21. Pay when parking in a blue -white zone as directed by the parking meter.
22. Change some part in the car (filter, strap, etc..) because the mechanic said it was old and due to fail.
23. Go through customs without declaring about goods I am bringing which are supposed to be taxed.
24. Report to social services about a child from the neighborhood that is being seriously neglected by his parents.
25. Not say anything when receiving too much change at the store.

Likert 7-point scale (Not likely at all=1; ***;*** *Very likely=7)*

3.12 Elaboration of possible outcomes

Generation/evaluation dimension

1. Before I act I consider what I will gain or lose in the future as a result of my actions
2. I try to anticipate as many consequences of my actions as I can.
3. Before I make a decision I consider all possible outcomes.
4. I always try to assess how important the potential consequences of my decisions might be.
5. I try hard to predict how likely different consequences are.
6. Usually I carefully estimate the risk of various outcomes occurring.

Positive outcome focus dimension

1. I keep a positive attitude that things always turn out all right.
2. I prefer to think about the good things that can happen rather than the bad.
3. When thinking over my decisions I focus more on their positive end results.

Negative outcome focus dimension

1. I tend to think a lot about the negative outcomes that might occur as a result of my actions.
2. I am often afraid that things might turn out badly.
3. When thinking over my decisions I focus more on their negative end results.
4. I often worry about what could go wrong as a result of my decisions.

Likert 7-point scale (Completely disagree=1; ***;*** *Completely agree=7*
